# Best practices and future challenges in the treatment of oral cancer

**DOI:** 10.1515/iss-2023-0031

**Published:** 2024-02-05

**Authors:** Juliane Kröplin, Jil-Charlot Reppenhagen

**Affiliations:** Department of Oral and Maxillofacial Surgery, University Medical Centre Rostock, Rostock, Germany

**Keywords:** squamous cell carcinoma, outcome quality, transoral robotic surgery, immunotherapy, intraoperative assessment

## Abstract

**Objectives:**

Oral cancer is among the most common tumour worldwide. Due to the anatomical peculiarities of the head and neck region, the treatment of oral cancer is a major challenge with regard to the preservation of aesthetics and function. The aim of the present study is to analyze currently practiced therapeutic strategies as well as current and future challenges in the therapy of oral cancer.

**Methods:**

A Pubmed-based selective literature search was performed considering literature predominantly from 2021 to 2022. Search terms were “oral cancer,” “oral cavity cancer,” and “head and neck cancer.”

**Results:**

Head and neck tumours are the seventh most common cancer worldwide. The suspected diagnosis of oral cancer is often made by outpatient dentists during routine examinations. With the outbreak of the 2020 COVID 19 pandemic, risk behaviour has changed with regard to the development and diagnosis of oral cancer. The gold standard of therapy is surgical resection. The need for adjuvant therapy measures depends on the histopathological TNM stage and other defined risk factors. Recurrences occur frequently and should be evaluated with regard to renewed surgical therapy. Future treatment strategies are aimed at early diagnosis, precision in resection, the use of digital technologies, and aspects of quality assurance. The economic importance in the treatment of oral cancer is currently given little consideration.

**Conclusions:**

The study presents a selective portfolio of treatment strategies currently practiced in Germany and in many parts of the world. In addition, future challenges in the therapy of oral cancer, in particular squamosa cell carcinoma, are presented.

## Introduction: etiology and epidemiology of oral cancer – current status and influencing factors

Tumours of the head and neck region are an underestimated global health risk. They are the seventh most common cancer worldwide. The incidence of the number of cases has increased significantly in recent years. Men are affected more than twice as often as women [1, 2]. More than 90 % of all malignancies of the oral cavity are squamous cell carcinomas. They are predominantly localized in the area of the tongue and the floor of the mouth. Tobacco and alcohol consumption are particular risk factors for the development of oral cavity carcinomas. In addition, oncogenic viruses, such as the human papilloma virus, are increasingly leading to an increase in the incidence of oropharyngeal cancer [1, 3].

## Influence of the COVID 19 pandemic

The influence of the COVID 19 pandemic on the behaviour of people towards risk factors for oral cancer is an aspect that has been analyzed and discussed in the context of scientific publications [4]. With the outbreak of the COVID 19 pandemic in 2020, the risk behaviour with regard to the development and diagnosis of oral cancer has changed. On the one hand, a changed lifestyle with increased consumption of tobacco and alcohol, poor nutrition and lack of oral hygiene became apparent. On the other hand, delays in diagnostics and therapy due to the lockdown measures in the context of the COVID 19 pandemic have been described. As a result, physicians and dentists are especially encouraged to be vigilant for potential behavioural changes in their patients. Education about risk factors that contribute to the development of oral cancer as well as regular routine check-ups with a special focus on the head and neck region are of particular importance [4].

## Diagnosis – strong partners in the battle against time

Malignancies in the head and neck regions represent a challenge for physicians in their treatment, especially in advanced stages. They require an interdisciplinary approach, including as essential surgery, radiotherapy and systematic therapy [1].

A decisive prognosis-relevant factor is the fastest possible confirmation of the diagnosis as well as immediate therapy. In fact, many affected patients do not consult a specialist until the disease has reached an advanced stage. As a result, patients are often confronted with limited therapeutic options. Late diagnosis thus not only reduces the chance of survival, but also limits the preservation of important everyday functions such as swallowing, speaking and eating [2].

The 5-year survival rate of patients in stage I–II is 70–90 %. In contrast, patients in stage III–IV have a significantly worse prognosis. Tumours that are discovered by chance during medical examinations are often smaller compared to those that are noticed by the patient [1].

Dentists therefore already play an important role in preventive early detection as the first point of contact for patients suspected of having a tumour. The majority of malignant changes in the oral mucosa are detected by dentists. Most commonly, patients complain of dysphagia, ear pain, hoarseness, mucosal irritation, and weight loss. However, routine screening is not mandatory for practitioners. The 2022 prospective observational study by Hertrampf et al. demonstrates the feasibility of such checks in private dental practices. In addition to the statutory dental check-up, the treating dentists are asked to perform a visual inspection of the oral cavity of the respective patients and to document it professionally by means of predefined questionnaires. The presumed diagnosis and localization are recorded. It should be noted that the visual examination of the oral mucosa, for the general early detection of malignant disease sites, is easy to perform, non-invasive, inexpensive and therefore uncomplicated for the patient [2].

Unlike breast, prostate and colorectal cancers, the detection of tumours in the oral and pharyngeal regions does not require extensive screening tests and can therefore be effectively mapped by the dentist during regular consultations [2]. Whether this will be effective in limiting the global problem is unclear at this time and will need to be investigated in further studies.

## Best treatment strategies – an interdisciplinary task

### Guideline-based therapy in primary cases

The National Comprehensive Care Network Clinical Practice Guideline for head and neck cancer is available for guideline-based therapy of squamous cell carcinoma of the oral cavity [5]. In addition, the S3 Guideline for the Diagnosis and Therapy of Oral Cavity Carcinoma of the Association of the Scientific Medical Societies (AWMF) has been available since 2021 [6]. The recommendations are based on tumour stage and tumour-specific characteristics.

### The primary therapy

The leading strategy is surgical resection. This provides the opportunity for histopathological characterization and accurate staging [3]. The pathological safety distance is at least 5 mm. In particular, smaller tumours (T1/T2) can be resected well and usually transorally. In addition to the resection of the primary tumour, a neck dissection is performed. Depending on the involvement of regional lymph nodes, this is either elective (END) or therapeutic (TND). Elective neck dissection generally covers levels I–III (II–IV for oropharyngeal tumours) and can be performed unilaterally for purely lateral tumours. Tumours crossing the midline or involvement of structures with bilateral lymphatic drainage pathways (e.g. the tongue) require bilateral elective neck dissection. In the case of manifest lymph node metastases, a modified radical neck dissection is usually performed including levels I–V with preservation of one or more non-lymphatic structures (n. accessorius, der v. jugularis, m. sternocleidomastoideus) – in the sense of a therapeutic neck dissection [1, 3].

Due to its complexity in terms of function and aesthetics, the head and neck region represents a major challenge for tumour surgery interventions. For the restoration of aesthetics and function after such interventions, reconstructive surgery, as another area of competence of the oral and maxillofacial surgeon, is of particular importance. Smaller defects can often be treated by local flap plasty. For larger defects, free tissue transfer is in the foreground. Depending on the defect situation, various microsurgical anastomosed grafts are used with very good results [1].

### Adjuvant therapy measures – radio(chemo)therapy

Smaller tumours without lymph node involvement (pN0) and without additional risk factors (positive (R1)/narrow resection margins, perineural invasion, invasion of lymphatic vessels, depth of invasion >10 mm) do not require adjuvant therapy. In case of larger tumours (T3/T4), lymph node metastasis and tumours with additional risk factors mentioned above, adjuvant radiotherapy is necessary. In special risk constellations – such as extranodal growth – this is supplemented by chemotherapy [1, 3].

### Further therapy options

Primary radiotherapy is a curative treatment option for smaller tumours. However, it should be noted that significantly higher radiation doses are required than in adjuvant therapy. The side effect profile of primary radiotherapy – such as dry mouth, radiation caries and the dreaded osteoradionecrosis of the jaw – is considerable, so that surgical therapy should be preferred whenever possible [3].

In cases of recurrence and/or advanced metastasis, where neither surgical resection nor radiotherapy is possible or not effective, immunotherapy is an option. In particular, PDL-1 inhibitors (e.g. pembrolizumab) and EGRF inhibitors are used. Depending on the PDL-1 expression, pembrolizumab alone (PDL-1 expression 1 % or more) or a combination therapy of pembrolizumab with chemotherapy (5-fluorouracil and platinum) is used [1].

In order to find the optimal therapeutic strategy, it is essential to inform the patient about the available options and to involve the patient in the decision-making process, taking into account the patient’s individual therapeutic goals and existing comorbidities.

### The importance of sentinel node biopsy for head and neck tumour surgery

If no lymph node metastases (cN0) manifest themselves clinically or radiologically, a sentinel node biopsy (SNB) can be performed accordingly. An interdisciplinary team consisting of oral and maxillofacial surgeons and otorhinolaryngologists has published consensus guidelines based on the 8th International Symposium on SNB in London: First of all, it is important to note that SNB is not a therapeutic option, but a diagnostic test. Patients with T1 and T2 tumours that can be removed by an oral approach at a sufficient safety distance (>5 mm at the pathological specimen) without requiring access to the neck for reconstruction are suitable. The most suitable radioisotope is technetium-99 m (99mTC). Optical tracers such as Indocyanine green can also be used. As shown in the European Node Trial, neck dissection could be prevented in 70 % of patients with T1-2 tumours of the oral cavity. However, it must be taken into account that up to 30 % of patients require a second procedure for therapeutic neck dissection. This is a circumstance that must be taken into account in differential therapy, especially in multimorbid patients. Randomized prospective studies on the comparability of SNB and END regarding survival outcome are currently not available. The risk of recurrence after SNB is comparable to elective neck dissection. A standardized follow-up protocol should be part of SNB therapy. Considering the above aspects, SNB allows individualized, resource-efficient patient care with reduced morbidity and excellent outcomes [7].

### The therapy of recurrences

Recurrences occur frequently. This drastically reduces the survival time of patients. Consequently, primary therapy must be selected in such a way that recurrences are avoided as best as possible. The greater the tumour stage of the primarius, the more likely it is that both recurrence-free survival and overall patient survival will be reduced. This is equally true for the presence of other risk factors such as the presence of lymph node metastases. Moreover, overall survival in patients with local recurrence is better than in those with regional recurrence.

The evidence regarding stringent therapy strategies in case of recurrence is limited. A retrospective study by Zittel et al., taking into account their own results and the available literature, came to the conclusion that surgical therapy of the recurrence should also be performed in previously irradiated patients, if possible. In the case of corresponding histopathological risk factors and a short latency until the recurrence (<12 months), adjuvant therapy in the sense of radio(chemo)therapy should also be performed [8].

## Future challenges and innovative strategies – perspectives in the treatment of oral cancer

### Precision and objectivity

The complete resection of a squamosa cell carcinoma with an adequate safety margin is the most important prognostic factor for the further course of the disease. However, it is essential for the functional and aesthetic outcome not to remove more tissue than necessary. A goal that is rarely achieved in this combination, as intraoperative feedback systems for safe orientation are limited. In addition to visual inspection, palpation and imaging visualization, there is also the possibility of intraoperative assessment of resection margins (IOARM). Barasso et al. conducted a review of literature in 2021 to investigate which forms of IOARM are currently used during surgical resection of oral cancer, their performance, and the clinical relevance of their use. The authors conclude that the use of IOARM improves the status of the resection margin. These are the specimen-guided IOARM and the defect-guided IOARM. The defect-guided method is based on frozen section analysis of tissue samples from the wound bed. The disadvantage of this method is that the margin value cannot be given with millimeter accuracy. In the specimen-guided method, the margins are assessed on the specimen by visual inspection and palpation. This is followed by vertical incisions with or without tissue sampling. This provides the surgeon with immediate feedback as to whether additional resection is required. According to the literature analyzed by Barasso et al. the specimen-driven IOARM is considered to be more efficient than the defect-driven IORAM.

As crucial disadvantages of both systems are the subjectivity as well as the fact that only a small part of the resection area can be assessed in a short period of time, which increases the error rate for samples. The aim of future support systems to improve the precision of resection margins should above all enable an objective and rapid assessment of the entire resection area [9].

### Robotics, AI and Big data – digital approaches to solutions in the treatment of oral cancer

In order to reduce the morbidity of oral cancer patients (OCP) and to improve the outcome, there are numerous digital solutions that are applied to various processes in the treatment of oral cancer patients – from prevention to tumour follow-up.

For example, in a retrospective analysis by Lin et al., a smartphone-based imaging diagnostic technique based on a deep learning algorithm was investigated. The goal was to automatically detect oral pathologies. The authors conclude that image acquisition of oral lesions combined with resampling of cases in the training set and the use of a deep learning network (HRNet), effectively improve the performance of the deep learning algorithm in detecting oral cancer.

Specifically, the machine learning (ML) approach – as a sub-field of artificial intelligence – enables insights to be gained from historical data and predictions to be derived with the help of what has been learned. The systematic review by Chiesa-Estomba et al., for example, uses various studies to show the potential of ML to significantly advance research in the field of oral cancer. The benefits are based on the use and training of ML models that can be continuously trained as more data become available. Both the management in the treatment of oral carcinoma patients and the predictions and forecasts could be significantly improved. The prerequisite for this is to improve and democratise the use of algorithms [10].

In the area of perioperative process optimisation, the focus is increasingly on minimally invasive surgery through robot-assisted surgery, in addition to digital planning systems [11]. Transoral robotic surgery (TORS) allows the surgeon to operate on tumours robotically. The primary objective is the more precise removal of tumours in order to spare the patient the adjuvant therapy measures which have many side effects. In the case of tumours that are difficult to access anatomically, it is also possible to avoid transcervical and transmandibular approaches and thus reduce postoperative morbidity [1]. Compared to other surgical fields – such as visceral surgery – the use of robot-assisted surgery in oral and maxillofacial surgery is still not widespread.

The digitalisation of various processes in the treatment of patients with oral cancer holds great potential that requires differentiated reflection and targeted further development.

### Outcome – a matter of quality

The treatment of oral cancer is sometimes accompanied by massive restrictions in terms of function and aesthetics. In addition to disease-specific survival and objective success criteria – such as R0 resection with an adequate safety margin – the quality of life of patients is increasingly coming into focus. According to the WHO, this is defined as “the individual perception of one’s own life situation in the context of the respective culture and value system and in relation to one’s own goals, expectations, assessment standards and interests”. Numerous analytical tools have been established for the assessment of oral health-related quality of life with the aim of standardisation and comparability of therapeutic measures. The meta-analysis by Yuwanati et al., taking into account the Oral Health Impact Profile (OHIP)-14 questionnaire, was able to show that oral health as well as oncological therapy can negatively influence the quality of life of patients with oral carcinoma. Compared to the normal population, OHRQoL is significantly worse in OCPs. Consequently, aspects of oral health and quality of life must be given greater consideration in order to be able to make even better therapy decisions in the future and to provide the best possible framework conditions for tumour aftercare [12].

### An economic evaluation

Medical studies related to oral cancer primarily consider clinical and epidemiological aspects. Evidence on the costs associated with the disease has been limited.

In a systematic review by Ribeiro-Rotta et al. from the year 2022, it was shown that currently available “cost of illness” studies, taking into account the entities lip carcinomas, oral cavity carcinomas and oropharyngeal carcinomas, are generally very heterogeneously designed and thus hardly comparable. Nevertheless, the review provides an interesting overview of the direct (medical and non-medical) and indirect costs (lost productivity and premature death) associated with the occurrence of oral cancer. Based on the available literature, it can be assumed that these costs are underestimated worldwide. Early detection of oral cavity cancer during routine dental examinations is a key factor in reducing costs. This is because advanced tumours require complex and expensive therapies, but are also associated with a reduced survival rate. For a better assessment of the actual economic significance, longitudinal incidence-based studies in particular should be carried out [13].

## Summary

Oral cancer is a globally underestimated health risk with increasing incidence. Risk factors are especially alcohol and tobacco consumption. A change in risk behaviour in this regard with the outbreak of the COVID-19 pandemic must be taken into account in routine examinations. Early diagnosis and therapy are prognosis-relevant and require good interdisciplinary and intersectoral cooperation. The leading therapeutic strategy is surgical resection. This also applies to the recurrence situation. Future treatment strategies are aimed at early diagnosis, precision in resection, the use of digital technologies and aspects of quality assurance. The economic significance in the therapy of oral cavity carcinomas is currently given little consideration.

## Conclusions

1. Oral cancer is a global health risk. Prevention and early diagnosis are becoming increasingly important on the basis of interdisciplinary and intersectoral cooperation.
2. Surgical therapy is the gold standard and should be evaluated as a treatment option in all stages – including a recurrence situation.
3. Aspects of structure, process and outcome quality are increasingly coming into focus in the treatment of head and neck tumours and should in particular also take into account the patients’ quality of life.
4. The possibilities of technical support systems are increasingly available and require innovative skills on the part of the treating surgeons.
5. The economic importance of the treatment of oral cancer is currently not given enough attention. Adequate management could make a positive contribution to the targeted distribution of necessary but limited resources.
